# Influence of Depolarizing Fields and Screening Effects on Phase Transitions in Ferroelectric Composites

**DOI:** 10.3390/ma11010085

**Published:** 2018-01-06

**Authors:** Boris Darinskii, Alexander Sidorkin, Alexander Sigov, Nadezhda Popravko

**Affiliations:** 1 Chemical Department, Voronezh State University, University sq. 1, 394018 Voronezh, Russia; darinskii@mail.ru; 2 Physical Department, Voronezh State University, University sq. 1, 394018 Voronezh, Russia; n-popravko@yandex.ru; 3 Institute of Physics and Technology, Moscow Technological University, Vernadsky Avenue 78, 119454 Moscow, Russia; assigov@yandex.ru

**Keywords:** ferroelectric nanocomposites, smart materials, phase transitions, dielectric properties

## Abstract

The temperature of the transition to the polar state in ferroelectric composites, representing spherical ferroelectric inclusions embedded in a dielectric matrix, under a depolarizing field effect is investigated. This temperature is determined both in the absence and presence of screening effects of the depolarizing field of the bound charges of spontaneous polarization at the inclusions surface. The absence case shows that the Curie point shift is determined by the ratio of the Curie constant of the ferroelectric inclusion to the permittivity of the matrix. Screening effects show that the transition temperature shift decreases through multiplying the value by a decreasing factor equal to the ratio of the screening length to the radius of the ferroelectric inclusion. Examples of the materials for the position of the Curie point on the temperature scale, largely determined by the tilting action of the depolarizing field and the compensating shielding effects, are given.

## 1. Introduction

The general increase in the requirements for functional opportunities of different devices has sharply increased the demands placed on their elemental base. Natural materials no longer satisfy the growing technological and operational requirements, due to the limited range of operating parameters, randomness of their characteristics, and the absence of possibilities for changing functional parameters. Artificial nanomaterials with controlled properties, as a result of the influence of nanoscale effects on the properties of materials, are a better fit for these purposes [1,2,3]. Ferroelectric nanocomposites are one such object, whose properties are extremely sensitive to size effects caused by the increased role of surface or boundary effects. The special sensitivity of these materials is caused by phase transitions, which increase the compliance of the structure to various impacts. 

Identification of linkages between various characteristics and the structure of these materials will allow researchers to find new ways of controlling their parameters, which is essential for practical materials science. The changes in the dielectric constant, including the dispersion of the permittivity of ferroelectric composites, their piezoelectric and pyroelectric properties, have been considered in several pieces of research [4,5,6,7,8,9,10,11,12,13,14].

One important characteristic with respect to the practical application of these materials is temperature range, where ferroelectric properties can be observed in the researched composites. Numerous experimental studies have shown that practically all studied ferroelectric nanocomposites exhibit a shift in the Curie temperature [15,16,17,18], both in the direction of high and low temperatures, in comparison with the corresponding homogeneous ferroelectrics. One factor that increases the Curie temperature (or Curie point) *T*_*C*_ of ferroelectric composites is the action of the internal field associated with fixation of polarization in the ferroelectric component by deformation fields arising at the boundary of various components [19,20]. Factors reducing *T*_*C*_ include correlation effects at the place of contact between the ferroelectric inclusion and the nonpolar phase, as well as the depolarizing fields arising near the surface of the ferroelectric inclusions. The effect on the Curie point in ferroelectric composites of the correlation interactions of the polar inclusion with the matrix, mechanical stresses and the depolarizing fields of the bound charges at the boundary of the ferroelectric inclusions has evaluated theoretically [21,22,23,24,25,26,27]. However, the effects of screening were not taken into consideration in these studies.

## 2. Materials and Methods

The present work calculates depolarizing fields arising near the boundaries of a spherical ferroelectric inclusion in an isotropic dielectric environment and evaluates the effect of these fields, and screening effects on the Curie point in the composites.

The theory of Landau–Ginzburg phase transitions was used to obtain the theoretical expression for the displacement of the Curie point in a ferroelectric sphere under bound charges by a spontaneous polarization on its surface and screening charges. The dielectric material around the ferroelectric sphere was assumed to be isotropic. It was supposed that the distance between the ferroelectric inclusions was sufficiently large, and thus the interaction between them was ignored. The comparison of theoretical results with experimental ones was made based on experimental data on the behavior of the Curie point in ferroelectric nanocomposites. Nanocrystalline cellulose with ferroelectric sodium nitrite particles and silica with triglycine sulfate particles were considered to be representative samples of these materials.

## 3. Results

### 3.1. The Considered Model and Basic Equations in the Absence of Screening

First, the effect of depolarizing fields near the surface of the ferroelectric inclusion on the Curie point, in the absence of screening effects, is considered. The thermodynamic potential of a sample containing a spherical ferroelectric single inclusion embedded in a linear dielectric, can be written as:

(1) $$F=a\;\frac{P^{2}}{2}\;\left(\frac{4\pi }{3}\;R^{3}\right)+\;\frac{E_{i}}{8\pi }\;\left(\frac{4\pi }{3}\;R^{3}\right)+\;\frac{\varepsilon }{8\pi }\;\int^{\text{​}}E^{2}\;d\vec{r},$$

where α is the expansion coefficient of thermodynamic potential into a series, in respect to polarization *P*; *R* is the radius of the ferroelectric inclusion; *E_i_* and *E* are the field inside and outside the ferroelectric inclusion, respectively; ε is the permittivity of the dielectric environment outside the inclusion.

To find the field inside the ferroelectric inclusion, the relationship between the electric field strength and the potential $\varphi =-E_{i}z$ (where z is the coordinate along the direction of the polar axis of the inclusion) is used. The expression for the potential outside the inclusion has the form characteristic of an electric dipole $\varphi =Az/r^{3}$. Equating both expressions according to the condition of continuity of the potential at the inclusion boundary, finds that: $A=-E_{i}R^{3}$. Considering the constant and using the continuity condition for the vector of electric induction

(2) $$E_{i}+4\pi P=\;-\varepsilon \;{\frac{\partial \varphi }{\partial z}|}_{R}=\;-\varepsilon A\;{\left(\frac{1}{R^{3}}-\;\frac{3z^{2}}{R^{5}}\right)|}_{R}=\;\frac{2A\varepsilon }{R^{3}}=\;-2\varepsilon E_{i}$$

finds:

(3) $$E_{i}=\;-\;\frac{4\pi P}{1+2\varepsilon }$$

To estimate the influence of these fields on the Curie point, a preliminary estimate of the last term in the expansion (Equation (1)) was made. Differentiating successively the potential $\varphi =Az/r^{3}$ with respect to individual coordinates, gives:

(4) $$E_{x}=\;\frac{3Azx}{r^{5}},\;\;E_{y}=\;\frac{3Azy}{r^{5}},\;\;E_{z}=\;-\frac{A^{2}}{r^{3}}+A\;\frac{3z^{2}}{r^{5}}$$

Thence:

(5) $${\left(\nabla \varphi \right)}^{2}=\;A^{2}\;\left(\frac{1}{r^{6}}+\;\frac{3z^{2}}{r^{8}}\right)$$

Respecting the obtained expression, as well as the expression for *A*, finds:

(6) $$\frac{\varepsilon }{8\pi }\int^{\text{​}}E^{2}d\vec{r}=\;\frac{\varepsilon \;4\pi \;P^{2}}{{\left(1+2\varepsilon \right)}^{2}}\;\left(\frac{4\pi }{3}\;R^{3}\right)$$

Thus, considering the contribution of the depolarizing fields, the thermodynamic potential *F* takes the form:

(7) $$F=\left(a\frac{P^{2}}{2}+\;\frac{{\left(4\pi P\right)}^{2}}{{\left(1+2\varepsilon \right)}^{2}}\;\frac{1}{8\pi }+\;\frac{4\pi P^{2}\varepsilon }{{\left(1+2\varepsilon \right)}^{2}}\right)\;\left(\frac{4\pi }{3}\;R^{3}\right)$$

Thus, the coefficient near the harmonic term is replaced by:

(8) $$a+\;\frac{2\pi }{\left(1+2\varepsilon \right)}$$

Therefore, the Curie point of the material decreases in accordance with the expression:

(9) $$T_{C}^{*}=T_{C}-\frac{2\pi }{\left(1+2\varepsilon \right)\;a_{0}}$$

where $T_{C}^{*}$ is Curie point taking into account the influence of depolarizing fields, *T_C_*—Curie point without account of these fields and $a_{0}$ is a derivative ∂a/∂T.

### 3.2. The Considered Model and Basic Equations in the Presence of Screening

**Figure 1:**
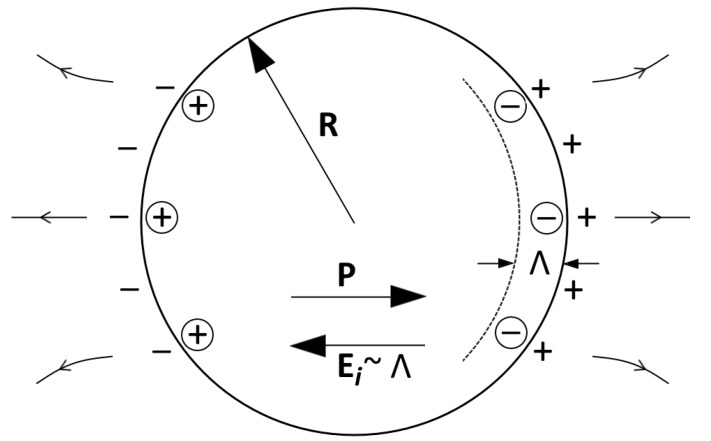
The field inside a sufficiently strongly screened ferroelectric inclusion: *R* is the inclusion radius; *P* is the spontaneous polarization of the inclusion; *E_i_* is the electric field strength inside the ferroelectric inclusion; Λ is the screening length.

The presence of a finite conductivity in ferroelectric composites leads to a screening of the depolarizing fields. Screening is dependent on the concentration of charged carriers, decreasing field of bound charge carriers at the boundary between the ferroelectric inclusion and the dielectric matrix. Consequently, a decrease in the shift of the Curie point in such ferroelectrics occurs. In the presence of the screening when the potential of the point charge q/r is replaced by q exp(−r/Λ)/r, for the potential of a ferroelectric inclusion outside its volume it is necessary to use the potential of a screened dipole (Figure 1):

(10) $$\varphi \left(\vec{r}\right)=\frac{Az}{r^{3}}\exp\left(-r/\Lambda \right)+\frac{Az}{r^{2}\Lambda }\exp\left(-r/\Lambda \right)$$

where Λ is a screening length. The condition of continuity of the potential on the boundary of the ferroelectric inclusion, finds:

(11) $$A=\frac{-R^{3}E_{i}}{\left(1+\frac{R}{\Lambda }\right)\exp\left(-R/\Lambda \right)}$$

Then, from the continuity condition for the electric induction vector at the inclusion boundary, the field inside the inclusion appears in the following form:

(12) $$E_{i}=-\frac{4\pi P}{\left[1+\frac{\varepsilon }{\left(1+R/\Lambda \right)}\left(2+\frac{2R}{\Lambda }+\frac{R^{2}}{\Lambda^{2}}\right)\right]}$$

where at Λ → ∞ in the absence of screening:

(13) $$E_{i}=-\frac{4\pi P}{\left(1+2\varepsilon \right)}$$

and at Λ << *R* with a sufficiently strong screening (Figure 1):

(14) $$E_{i}=-\frac{4\pi P\Lambda }{\varepsilon R}$$

To estimate the effect of depolarizing fields on the Curie point position, first calculate their contribution to the thermodynamic potential (Equation (1)). Obtain the electric field components outside the ferroelectric sphere by differentiating the electric potential (Equation (10)) with different coordinates:

(15) $$\begin{array}{c} E_{x}=\;\left(\frac{3Axz}{r^{5}}+\;\frac{3Axz}{r^{4}\Lambda }+\;\frac{Axz}{r^{3}\Lambda^{2}}\right)\exp\left(-r/\Lambda \right),\\ E_{y}=\;\left(\frac{3Ayz}{r^{5}}+\;\frac{3Ayz}{r^{4}\Lambda }+\;\frac{Ayz}{r^{3}\Lambda^{2}}\right)\exp\left(-r/\Lambda \right),\\ E_{z}=\;\left(\frac{3Az^{2}}{r^{5}}+\;\frac{3Az^{2}}{r^{4}\Lambda }-\;\frac{A}{r^{3}}+\;\frac{Az^{2}}{r^{3}\Lambda^{2}}-\;\frac{A}{r^{2}\Lambda }\right)\exp\left(-r/\Lambda \right) \end{array}$$

Generally, the contribution of these fields to the thermodynamic potential (Equation (1)) looks cumbersome. It can be presented more clearly with sufficiently strong screening:

(16) $${\left(\nabla \varphi \right)}^{2}\;\approx \;\left(\frac{A^{2}x^{2}z^{2}}{r^{6}\Lambda^{4}}+\;\frac{A^{2}y^{2}z^{2}}{r^{6}\Lambda^{4}}+\;\frac{A^{2}z^{4}}{r^{6}\Lambda^{4}}\right)\exp\left(-2r/\Lambda \right)=\;\frac{A^{2}z^{2}}{r^{4}\Lambda^{4}}\exp\left(-2r/\Lambda \right)$$

Regarding the expression found for $E^{2}$, the thermodynamic potential (Equation (1)) can be rewritten as:

(17) $$F=a\;\frac{P^{2}}{2}\;\left(\frac{4\pi }{3}\;R^{3}\right)+\;\frac{E_{i}}{8\pi }\;\left(\frac{4\pi }{3}\;R^{3}\right)+\;\frac{\varepsilon }{8\pi }\;\frac{R^{3}}{2\Lambda^{3}}\;\frac{E_{i}^{2}}{{\left(1+\;r/\Lambda \right)}^{2}}\;\left(\frac{4\pi }{3}\;R^{3}\right)$$

Substituting the field $E_{i}$ in the form Equation (14):

(18) $$F=\;\left(a\frac{P^{2}}{2}+\;\frac{2\pi P^{2}}{\varepsilon }\;\frac{\Lambda }{R}\right)\left(\frac{4\pi }{3}R^{3}\right)$$

Therefore, the decrease of the Curie point of the ferroelectric inclusion with a sufficiently strong screening is determined by the expression:

(19) $$T_{C}^{*}=\;T_{C}-\;\frac{2\pi }{\varepsilon a_{0}}\;\frac{\Lambda }{R}\;=\;T_{C}-\;\frac{C}{\varepsilon }\frac{\Lambda }{R}$$

where *C* is the Curie–Weiss constant.

## 4. Discussion

### Theoretical Estimates and Comparison with Experiment

According to Equation (9), the shift of the Curie point by an unscreened depolarizing field is approximately equal to the ratio C/ε. Thus, an ordinary Curie–Weiss constant *C* (for example, in materials with a second-order phase transition *C* is about 10^3^) and the dielectric constant ε (which is about 10 for dielectric matrix), the shift of the Curie point has a large magnitude of hundreds of degrees. The screening reduces the shift of the Curie point and makes it more real. Indeed, according to Equation (19) with screening, this shift is reduced by a factor equal to the ratio of the screening length to the radius of the ferroelectric inclusion. Under normal conditions, the screening length $\Lambda =\sqrt{\left(kT/\left(4\pi ne^{2}\right)\right)}$ (where *T* is the absolute temperature, n is the carrier concentration, *e* is the electron charge) is about 10^−7^ cm, essentially, the order of the unit cell constant. Regarding *R* ~ 10^−6^ cm, the decreasing factor is 0.1; thus, the shift of the Curie point becomes equal to approximately ten degrees. This, in order of magnitude, coincides with the Curie point shifting, as observed experimentally in ferroelectric composites.

The position of the Curie point in real ferroelectric composites is determined by the effect of several factors; therefore, it is difficult to define the influence of the depolarizing fields. The tipping action of the depolarizing field is usually superimposed by the tightening effect of the internal field of any nature. This displaces the Curie point into the high-temperature region. Consequently, the Curie point in ferroelectric composites is usually shifted to high temperatures. The tightening effect on the position of the Curie point is determined by the interaction of an inclusion with the environment, which is usually strong. Nevertheless, there is an example where such interaction is weakened, enabling the real influence of the depolarizing field to be observed.

The authors suppose that the example of such material can be a composite of nanocrystalline cellulose with ferroelectric sodium nitrite, for which the Curie point is displaced approximately 40 degrees lower on the temperature scale relative to bulk sodium nitrite [28]. This behavior of the composite is conditional on the interaction of the ferroelectric inclusion and the matrix being weakened or completely absent. The absence of hydrogen bonds, which ensure a strong interaction of the matrix with introduced material (as in a composite of nanocrystalline cellulose with triglycine sulfate), may be the reason for this occurring.

Another demonstration of depolarizing fields and screening effects’ influence is the behavior of the mixture composite triglycine sulfate and silica [29]. The measurements of dielectric permittivity for the just-prepared samples of this composite show a Curie point of about 100 °C. This is the result of the influence of all factors, which include the internal field that tightens the polarization in the paraphase, the upsetting effect of the depolarizing field, and the correlation effects. Note that screening effects develop quite slowly and, therefore, most likely do not add to these factors. Following 1000 h of exposure in room conditions, (temperature is about 300 K, pressure is 760 mmHg and relative humidity is carefully controlled between 35% and 45%), the Curie point in this composite is displaced further to a high temperature region for about 35 K. This increase might be related to the screening of charges of spontaneous polarization at the place of contact of a ferroelectric material with dielectric inclusions that passes during the aging of the samples in the ferroelectric phase. This screening destroys the action of unscreened spontaneous polarization charges in freshly prepared samples. Therefore, it can be assumed that displacement of the phase transition temperature under the field of spontaneous polarization charges is presumably equal to the same 35 K, but in low temperatures.

## 5. Conclusions

The studies of depolarizing field effects on the transition temperature in ferroelectric composites, with spherical ferroelectric inclusions embedded in the dielectric matrix, demonstrate that, in the absence of screening effects, the decrease of the Curie point in composites compared with bulk materials is determined by the ratio of the ferroelectric inclusion Curie constant to the permittivity of the matrix. The *T_C_* shift in these composites with screening is reduced by multiplying the above value by a decreasing factor equal to the ratio of the screening length to the radius of the ferroelectric inclusion.

Examples of the influence of depolarizing fields in the presence and absence of screening effects can be a mixed silica–triglycine sulfate composite and a nanocrystalline cellulose–sodium nitrite matrix composite [28,29]. This shows that the theoretical estimates of changes in temperature of phase transitions in ferroelectric composites, considering the influence of depolarizing fields and screening effects near the surface of the ferroelectric inclusion, are generally consistent with the experimental observation results for the Curie point position in ferroelectric composites.
